# Fixed-Time Synchronization Control of Delayed Dynamical Complex Networks

**DOI:** 10.3390/e23121610

**Published:** 2021-11-30

**Authors:** Mei Liu, Binglong Lu, Zhanfeng Li, Haijun Jiang, Cheng Hu

**Affiliations:** 1 School of Mathematics and Statistics, Zhoukou Normal University, Zhoukou 466001, China; lubinglong1981@163.com (B.L.); lihui007309420@163.com (Z.L.); 2 College of Mathematics and System Sciences, Xinjiang University, Urumqi 830046, China; jianghaijunxju@163.com (H.J.); wacheng2003@163.com (C.H.)

**Keywords:** complex networks, feedback control, adaptive control, fixed-time synchronization, delay

## Abstract

Fixed-time synchronization problem for delayed dynamical complex networks is explored in this paper. Compared with some correspondingly existed results, a few new results are obtained to guarantee fixed-time synchronization of delayed dynamical networks model. Moreover, by designing adaptive controller and discontinuous feedback controller, fixed-time synchronization can be realized through regulating the main control parameter. Additionally, a new theorem for fixed-time synchronization is used to reduce the conservatism of the existing work in terms of conditions and the estimate of synchronization time. In particular, we obtain some fixed-time synchronization criteria for a type of coupled delayed neural networks. Finally, the analysis and comparison of the proposed controllers are given to demonstrate the validness of the derived results from one numerical example.

## 1. Introduction

Complex network is a model that describes the relationship between nature and human society. It is a collection of many nodes and edges between connecting nodes. Nodes are used to represent different individuals in the real system, such as organisms in the food chain network and individuals in the social network, while edges are used to represent the relationship between individuals, such as the predator-prey relationship between organisms, friendship between people, etc. In fact, complex networks are ubiquitous in the real world, such as neural networks [1,2] formed by the interaction of a large number of neuronal cells through neural fibers, computer networks [3] formed by the interconnection of autonomous computers through communication media, and similar social relationship networks [4], transportation networks [5], power networks [6], robot networks [7,8,9], regulation networks [10], etc.

In the research of complex network dynamic behavior and group behavior, synchronization behavior has attracted more and more experts and scholars’ attention because of its important practical significance and universality. In short, synchronization is a kind of overall coordinated dynamic behavior formed by external force or mutual coupling between dynamic systems. Through long-term observation and research, it is found that synchronization phenomena widely exist in human society and nature, such as the synchronization of cardiomyocytes and brain nerve cells [11,12,13], the asymptotic synchronization of audience applause frequency in the theater, the synchronous luminescence of fireflies, etc. At present, the synchronization behavior analysis of complex networks has become an important research hotspot [14,15,16,17].

In the current research results of complex network synchronization theory, synchronization generally needs infinite time control to achieve. However, in practice, infinite time control is unreasonable. In terms of the perspective of cost, infinite time control and high gain control become uneconomic. For a real complex network, we often hope to realize the synchronization in a limited time as soon as possible by controlling the cost as low as possible. For example, in chaotic secure communication networks, based on security considerations, the network is often required to achieve fast synchronization in a limited time, so as to ensure that the decoded information is sent in a short time without disclosure. At present, some researchers have begun to study the finite-time synchronization of complex networks and obtained some valuable results [18,19,20,21].

In the theoretical analysis of finite-time synchronization, a key problem is how to effectively estimate the synchronization settling time and find an upper bound. Generally speaking, the synchronization settling time and its estimation of the networks under consideration depend on the initial value. When judging whether the solutions starting from multiple different initial values converge in finite-time in practical problems, it needs to calculate the settling time for many times. In order to overcome the inconvenience and limitation caused by the correlation between settling time estimation and initial value, Polyakov proposed the concept of fixed-time stability of dynamic system in 2012 and gave relevant criteria [22], which provides a theoretical basis for analyzing the fixed-time synchronization problem of complex networks. Therefore, the study of fixed-time synchronization in complex networks is a problem worthy of consideration by many scholars [23,24,25,26,27,28,29,30,31,32,33,34,35].

As we know, in the process of information transmission and spreading, the communication delay is a typical phenomenon and may result in oscillation and instability dynamic behaviors. Hence, it is necessary to study the influence of time delay on network’ dynamic behavior. At present, many scholars are studying time-delay dynamic networks [28,29,30]. In reference [28], Wang et al. studied global synchronization in fixed-time for semi-Markovian switching complex dynamical networks with hybrid couplings and time-varying delays. In reference [29], Cao et al. discussed fixed-time synchronization of delayed memristor-based recurrent neural networks. In reference [30], Chen et al. analyzed fixed-time synchronization of inertial memristor-based neural networks with discrete delay. However, as far as I am concerned, the fixed-time synchronization problem for dynamical delayed complex networks via feedback control and adaptive control receives litter attention at present. Hence, this challenging question should be solved in this paper.

Motivated by the above discussion, fixed-time synchronization of complex dynamical delayed networks will be investigated via feedback control strategy and adaptive control strategy. Fixed-time synchronization criteria and some corollaries are obtained in our paper which are very verifiable and useful in application. Compared with the previous results, our main results are more general and less conservative. The innovations of the paper are at least the following aspects.

1. The problem synchronization of complex networks has been studied in references [14,18,19,27]. However, the models they considered do not have time delays. In view of the importance of time delay, the model considered in this paper is complex network model with time delay.

2. A new theorem is used to realize fixed-time synchronization of complex networks. Unlike reference [32], the upper bound of stability time is, respectively, estimated for cases a<0 and a>0 and two different formulas of the estimate were obtained. A unified form of the estimate is derived for the cases a<0 and a>0 in this paper.

3. By designing adaptive controller and discontinuous feedback controller, fixed-time synchronization can be realized through regulating the main control parameter. As corollaries, some fixed-time synchronization criteria for a type of coupled delayed neural networks are obtained which are considered in references [18,19,27,32].

The remainder of this paper is structured as follows. In Section 2, a class of dynamical complex networks with delay and preliminaries are given. Fixed-time synchronization of the considered model under the feedback control strategy and adaptive control strategy is investigated based delay in Section 3. In Section 4, the effectiveness and feasibility of the developed methods are presented by one numerical example. Finally, some conclusions are obtained in Section 5.

Notations: Let $R^{N}$ be the space of *N*-dimensional real column vectors. For $x={\left(x_{1},\cdots ,x_{N}\right)}^{T}\in R^{N}$, ∥x∥ denotes a vector norm defined by $∥x∥={\left(\sum_{i=1}^{N}x_{i}^{2}\right)}^{\frac{1}{2}}$. We define $\left\|\phi \right\|={\left[\sum_{i=1}^{N}|\phi_{i}\left(t\right){|}^{2}\right]}^{\frac{1}{2}},$ for all $\phi ={\left(\phi_{1}\left(t\right),\phi_{2}\left(t\right),...,\phi_{N}\left(t\right)\right)}^{T}\in C\left(\left[-\tau ,0\right],R^{N}\right)$, which denotes the Banach space of all continuous functions mapping $\left[-\tau ,0\right]\to R^{N}$. Define $e\left(t\right)={\left(e_{1}^{T}\left(t\right),e_{2}^{T}\left(t\right),...,e_{N}^{T}\left(t\right)\right)}^{T}$, $|e_{i}{\left(t\right)|}^{\mu }={\left(|e_{i}^{1}{\left(t\right)|}^{\mu },|e_{i}^{2}{\left(t\right)|}^{\mu },...,{\left|e_{i}^{n}\left(t\right)\right|}^{\mu }\right)}^{T},\;\mathrm{sign}\left(e_{i}\left(t\right)\right)=\mathrm{diag}\left(\mathrm{sign}\left(e_{i}^{1}\left(t\right)\right),\mathrm{sign}\left(e_{i}^{2}\left(t\right)\right),...,\mathrm{sign}\left(e_{i}^{n}\left(t\right)\right)\right)$. $I_{N}$ is the identity matrix with *N* dimensions. $\lambda_{\max}\left(A\right)$ is the maximum eigenvalues of matrix *A*.

## 2. Preliminaries

Consider a general complex network involving *N* linearly identical nodes which is depicted by

(1) $$\begin{array}{ccc} {\dot{x}}_{i}\left(t\right) & = & f\left(t,x_{i}\left(t\right),x_{i}\left(t-\tau \left(t\right)\right)\right)+c\sum_{j=1,j\neq i}^{N}b_{ij}\Gamma \left(x_{j}\left(t\right)-x_{i}\left(t\right)\right),\\ \; & \; & \;\;\;\;i\in I=\{1,2,...,N\}, \end{array}$$

where $x_{i}\left(t\right)={\left(x_{i}^{1}\left(t\right),x_{i}^{2}\left(t\right),...,x_{i}^{n}\left(t\right)\right)}^{T}\in R^{n}$ represents the state variable of the *i*th node, $f:R\times R^{n}\times R^{n}\to R^{n}$ is a continuous function governing the dynamics of isolated nodes, the time-varying delay τ(t) denotes the internal delay, the constant c>0 is the coupling strength, $\Gamma =\mathrm{diag}\{\gamma_{1},\gamma_{2},...,\gamma_{n}\}$ is the inner connecting matric with $\gamma_{i}>0$, and $B={\left(b_{ij}\right)}_{N\times N}$ stands for the inner topology structure and satisfies the following conditions [32,33].

(2) $$\begin{array}{c} b_{ij}\geq 0,\;\;i\neq j,\;\;b_{ii}=-\sum_{j=1,j\neq i}^{N}b_{ij}. \end{array}$$

Based on the condition (2), network model (1) can be rewritten as follows:

(3) $$\begin{array}{ccc} {\dot{x}}_{i}\left(t\right) & = & f\left(t,x_{i}\left(t\right),x_{i}\left(t-\tau \left(t\right)\right)\right)+c\sum_{j=1}^{N}b_{ij}\Gamma x_{j}\left(t\right),\;\;\;\;i\in I. \end{array}$$

**Remark** **1.** *In reference [33], the author studies the following model:*

$$\begin{array}{ccc} {\dot{x}}_{i}\left(t\right) & = & f\left(t,x_{i}\left(t\right)\right)+c\sum_{j=1}^{N}b_{ij}\Gamma x_{j}\left(t\right),\;\;i\in I. \end{array}$$

*By referring the paper of reference [33], we investigate a general complex networks model with time delay which is described as follows:*

$$\begin{array}{ccc} {\dot{x}}_{i}\left(t\right) & = & f\left(t,x_{i}\left(t\right),x_{i}\left(t-\tau \left(t\right)\right)\right)+c\sum_{j=1}^{N}b_{ij}\Gamma x_{j}\left(t\right),\;\;i\in I, \end{array}$$

*where $f:R\times R^{n}\times R^{n}\to R^{n}$ is a continuous nonlinear function governing the dynamics of isolated nodes, and $c\sum_{j=1}^{N}b_{ij}\Gamma x_{j}\left(t\right)$ is a coupling term, which represents the linear identical coupling between each node of network.*

**Definition**  **1** (reference [34])**.** *The hyperplane*

$$\Lambda =\left\{{\left(x_{1}^{T},...,x_{N}^{T}\right)}^{T}\in R^{nN},\;x_{1}\left(t\right)=\cdot \cdot \cdot =x_{N}\left(t\right)=\Pi \left(t\right)\in R^{n}\right\}$$

*is said to be the synchronization manifold of (3), and Π(t) is called the synchronous state of the network (3).*

*Evidently, according to (3), Π(t) satisfies the following dynamic equation:*

(4) $$\begin{array}{ccc} \dot{\Pi }\left(t\right) & = & f(t,\Pi (t),\Pi (t-\tau (t))),\;i\in I. \end{array}$$

*The dynamic evolution Π(t) satisfying (3) with condition value $\varphi \in C(\left[-\tau ,0\right],R^{n})$ is called the synchronization state, which may be an equilibrium point, a periodic orbit, or ever a chaotic attractor.*

**Assumption**  **1** (reference [15])**.** *For the vector-valued function f(t,x(t),x(t−τ(t))), suppose the uniform semi-Lipschitz condition with respect to the time t holds, i.e., for any $x\left(t\right),\;y\left(t\right)\in R^{n}$, there exist positive constants $l_{1}>0$ and $l_{2}>0$ such that*

$$\begin{array}{c} \;\;\;\;{\left(x(t)-y(t)\right)}^{T}\left(f(t,x(t),x(t-\tau (t)))-f(t,y(t),y(t-\tau (t)))\right)\\ \leq l_{1}{\left(x(t)-y(t)\right)}^{T}\left(x(t)-y(t)\right)\;+\;l_{2}{\left(x(t-\tau (t))-y(t-\tau (t))\right)}^{T}\left(x(t-\tau (t))-y(t-\tau (t))\right). \end{array}$$

**Definition**  **2** (reference [26])**.** Dynamic systems (3) and (4) are said to realize fixed-time synchronized if, for any solutions of the models (3) and (4) represented by $x_{i}\left(t\right)={\left(x_{i}^{1},x_{i}^{2},...,x_{i}^{n}\right)}^{T}$ and $\Pi \left(t\right)={\left(\Pi_{1},\Pi_{2},...,\Pi_{n}\right)}^{T}$ started from different initial states $\varphi_{i}$ and ϕ, there is a time point $T^{*}\left(\varphi_{i},\phi \right)$ such that

$$\lim_{t\to T^{*}\left(\varphi_{i},\phi \right)}∥\Pi \left(t\right)-x_{i}\left(t\right)∥=0,\;\;∥\Pi \left(t\right)-x_{i}\left(t\right)∥=0,\;\;t\geq T^{*}\left(\varphi_{i},\phi \right),$$

and another time point $T_{\max}$ can be found such that $T^{*}\left(\varphi_{i},\phi \right)\leq T_{\max}$ for any $\varphi_{i},\phi \in C\left(\left[-\tau ,0\right],R^{N}\right)$, and

$$T\left(\varphi_{i},\phi \right)=\inf\{T^{*}\left(\varphi_{i},\phi \right)\geq 0:∥\Pi \left(t\right)-x_{i}\left(t\right)∥=0,t\geq T^{*}\left(\varphi_{i},\phi \right)\}$$

*is said to the synchronized settling time.*

**Lemma**  **1** (reference [18])**.** *Let $a_{1},a_{2},...,a_{n},\;\omega >1$ be positive numbers, and $0\leq r_{1}<r_{2}$; then,*

$${\left(\sum_{i=1}^{n}a_{i}^{r_{2}}\right)}^{\frac{1}{r_{2}}}\leq {\left(\sum_{i=1}^{n}a_{i}^{r_{1}}\right)}^{\frac{1}{r_{1}}},\;\;\sum_{i=1}^{n}a_{i}^{\omega }\geq n^{1-\omega }{\left(\sum_{i=1}^{n}a_{i}^{2}\right)}^{\frac{\omega }{2}}.$$

*Especially, if we select $r_{2}=1$ and $r_{1}=\frac{1+\mu }{2}\left(0\leq \mu <1\right)$, then, $0<r_{1}<r_{2}$, and the following inequality holds:*

$${\left(a_{1}+a_{2}+\cdot \cdot \cdot +a_{n}\right)}^{\frac{1+\mu }{2}}\leq a_{1}^{\frac{1+\mu }{2}}+a_{2}^{\frac{1+\mu }{2}}+\cdot \cdot \cdot +a_{n}^{\frac{1+\mu }{2}}.$$

**Lemma**  **2** (reference [15])**.** *If Y and Z are real matrices with appropriate dimensions, then, there exists a positive constant ς>0 such that*

$$Y^{T}Z+Z^{T}Y\leq \varsigma Y^{T}Y+\frac{1}{\varsigma }Z^{T}Z.$$

**Lemma**  **3** (reference [26])**.** *If there exists a nonzero real number a, positive numbers b and c,β∈[0,1),θ>1 satisfying a<min{b,c} such that*

$$\dot{V}\left(x\left(t\right)\right)\leq aV\left(x\left(t\right)\right)-bV^{\beta }\left(x\left(t\right)\right)-cV^{\theta }\left(x\left(t\right)\right),\;\;x\left(t\right)\in R^{n}\setminus \left\{0\right\},$$

*then, V(x(t))=0 and x(t)=0 for any t≥T, where*

$$T\leq T^{*}=\left\{\begin{array}{c} \frac{1}{a(1-\beta )}\ln\left(\frac{b}{b-a}\right)+\frac{1}{a(\theta -1)}\ln\left(\frac{c}{c-a}\right),\;\;a\neq 0,\\ \frac{1}{b(1-\beta )}+\frac{1}{c(\theta -1)},\;\;a=0. \end{array}\right.$$

In order to make the states of network (3) fixed-time synchronize with Π(t), then, we have the following controlled delayed dynamical network:

(5) $${\dot{x}}_{i}\left(t\right)=f\left(t,x_{i}\left(t\right),x_{i}\left(t-\tau \left(t\right)\right)\right)+c\sum_{j=1}^{N}b_{ij}\Gamma x_{j}\left(t\right)+u_{i}\left(t\right),$$

where $u_{i}\left(t\right)$ is an appropriate control gain.

## 3. Fixed-Time Synchronization

In this section, the coupling networks model with time-varying delay will be investigated. With the help of Lemma 3, how to design suitable $\eta_{1},\;\eta_{2},\;\eta_{3},\;\eta_{4}$ and μ,δ, such that the delayed controlled network (5) can achieve fixed-time synchronization will be shown. The main results are given as follows.

### 3.1. Discontinuous State Feedback Control

In order to get the main results, we design the following state feedback control.

(6) $$u_{i}\left(t\right)=\begin{array}{c} -\eta_{1}e_{i}\left(t\right)-\eta_{2}e_{i}\left(t-\tau \left(t\right)\right)-\eta_{3}\mathrm{sign}\left(e_{i}\left(t\right)\right)|e_{i}{\left(t\right)|}^{\mu }-\eta_{4}\mathrm{sign}\left(e_{i}\left(t\right)\right){\left|e_{i}\left(t\right)\right|}^{\delta }, \end{array}$$

where $\eta_{1},\;\eta_{2},\;\eta_{3},\;\eta_{4}>0$ are the control gains, and μ satisfies 0≤μ<1, δ satisfies δ>1.

Let $e_{i}\left(t\right)={\left(e_{i}^{1}\left(t\right),e_{i}^{2}\left(t\right),...,e_{i}^{n}\left(t\right)\right)}^{T}=x_{i}\left(t\right)-\Pi \left(t\right)\;\left(i\in I\right)$ be synchronization errors. According to the control law (6), the error dynamical network is then governed as follows:

(7) $$\begin{array}{ccc} {\dot{e}}_{i}\left(t\right) & = & \tilde{f}\left(t,x_{i},\Pi ,x_{i}^{\tau },\Pi^{\tau }\right)+c\sum_{j=1}^{N}b_{ij}\Gamma e_{j}\left(t\right)-\eta_{1}e_{i}\left(t\right)-\eta_{2}e_{i}\left(t-\tau \left(t\right)\right)\\ \; & \; & -\eta_{3}\mathrm{sign}\left(e_{i}\left(t\right)\right)|e_{i}{\left(t\right)|}^{\mu }-\eta_{4}\mathrm{sign}\left(e_{i}\left(t\right)\right){\left|e_{i}\left(t\right)\right|}^{\delta }, \end{array}$$

where $\tilde{f}\left(t,x_{i},\Pi ,x_{i}^{\tau },\Pi^{\tau }\right)=f\left(t,x_{i}\left(t\right),x_{i}\left(t-\tau \left(t\right)\right)\right)-f\left(t,\Pi (t),\Pi (t-\tau (t))\right)$.

**Theorem** **1.** *Under Assumption 1 and the controller (6), if*

$$p_{1}<\min\left\{2\eta_{3},\;2\eta_{4}{\left(nN\right)}^{-\delta }\right\},\;2l_{2}-\frac{\eta_{2}}{\varsigma }\leq 0,$$

*where $p_{1}=\lambda_{\max}\left(\left(2l_{1}-2\eta_{1}-\eta_{2}\varsigma \right)I_{N}+2c\gamma_{k}\frac{B^{T}+B}{2}\right),$ then, the controlled delayed dynamical network (5) is the fixed-time synchronized. Moreover, the synchronized settling time is estimated by*

$$T\leq T_{1}^{*}=\frac{1}{p_{1}\left(1-\frac{1+\mu }{2}\right)}\ln\left(\frac{2\eta_{3}}{2\eta_{3}-p_{1}}\right)+\frac{1}{p_{1}\left(\frac{1+\delta }{2}-1\right)}\ln\left(\frac{2\eta_{4}{\left(nN\right)}^{-\delta }}{2\eta_{4}{\left(nN\right)}^{-\delta }-p_{1}}\right).$$

**Proof.**  Construct the Lyapunov function as

(8) $$\begin{array}{c} V\left(t\right)=\sum_{i=1}^{N}e_{i}^{T}\left(t\right)e_{i}\left(t\right). \end{array}$$

Then, its derivative along with solutions of (7) can be given as below.

(9) $$\begin{array}{ccc} \dot{V}\left(t\right) & = & 2\sum_{i=1}^{N}e_{i}^{T}\left(t\right)[\tilde{f}\left(t,x_{i},\Pi ,x_{i}^{\tau },\Pi^{\tau }\right)+c\sum_{j=1}^{N}b_{ij}\Gamma e_{j}\left(t\right)-\eta_{1}e_{i}\left(t\right)\\ & \; & -\eta_{2}e_{i}\left(t-\tau \left(t\right)\right)-\eta_{3}\mathrm{sign}\left(e_{i}\left(t\right)\right)|e_{i}{\left(t\right)|}^{\mu }-\eta_{4}\mathrm{sign}\left(e_{i}\left(t\right)\right){\left|e_{i}\left(t\right)\right|}^{\delta }] \end{array}$$

$$\begin{array}{ccc} & \leq & 2l_{1}\sum_{i=1}^{N}e_{i}^{T}\left(t\right)e_{i}\left(t\right)+2l_{2}\sum_{i=1}^{N}e_{i}^{T}\left(t-\tau \left(t\right)\right)e_{i}\left(t-\tau \left(t\right)\right)+2c\sum_{i=1}^{N}\sum_{j=1}^{N}e_{i}^{T}\left(t\right)\\ & & \times b_{ij}\Gamma e_{j}\left(t\right)-2\eta_{1}\sum_{i=1}^{N}e_{i}^{T}\left(t\right)e_{i}\left(t\right)-2\eta_{2}\sum_{i=1}^{N}e_{i}^{T}\left(t\right)e_{i}\left(t-\tau \left(t\right)\right)\\ & & -2\eta_{3}\sum_{i=1}^{N}e_{i}^{T}\left(t\right)\mathrm{sign}\left(e_{i}\left(t\right)\right)|e_{i}{\left(t\right)|}^{\mu }-2\eta_{4}\sum_{i=1}^{N}e_{i}^{T}\left(t\right)\mathrm{sign}\left(e_{i}\left(t\right)\right){\left|e_{i}\left(t\right)\right|}^{\delta }\\ & \leq & \sum_{i=1}^{N}\sum_{k=1}^{n}e_{i}^{k}\left(t\right)\left(2l_{1}-2\eta_{1}-\eta_{2}\varsigma \right)e_{i}^{k}\left(t\right)+2c\sum_{i=1}^{N}\sum_{j=1}^{N}\sum_{k=1}^{n}\gamma_{k}e_{i}^{k}\left(t\right)\\ & & \times \frac{b_{ji}+b_{ij}}{2}e_{j}^{k}\left(t\right)+\left(2l_{2}-\frac{\eta_{2}}{\varsigma }\right)\sum_{i=1}^{N}e_{i}^{T}\left(t-\tau \left(t\right)\right)e_{i}\left(t-\tau \left(t\right)\right)\\ & & -2\eta_{3}\sum_{i=1}^{N}e_{i}^{T}\left(t\right)\mathrm{sign}\left(e_{i}\left(t\right)\right)|e_{i}{\left(t\right)|}^{\mu }-2\eta_{4}\sum_{i=1}^{N}e_{i}^{T}\left(t\right)\mathrm{sign}\left(e_{i}\left(t\right)\right){\left|e_{i}\left(t\right)\right|}^{\delta }\\ & \leq & \sum_{k=1}^{n}{\left({\tilde{e}}^{k}\left(t\right)\right)}^{T}\left[\left(2l_{1}-2\eta_{1}-\eta_{2}\varsigma \right)I_{N}+2c\gamma_{k}\frac{B^{T}+B}{2}\right]{\tilde{e}}^{k}\left(t\right)\\ & & -2\eta_{3}\sum_{i=1}^{N}e_{i}^{T}\left(t\right)\mathrm{sign}\left(e_{i}\left(t\right)\right)|e_{i}{\left(t\right)|}^{\mu }-2\eta_{4}\sum_{i=1}^{N}e_{i}^{T}\left(t\right)\mathrm{sign}\left(e_{i}\left(t\right)\right){\left|e_{i}\left(t\right)\right|}^{\delta }, \end{array}$$

where ${\tilde{e}}^{k}={\left(e_{1}^{k},e_{2}^{k},...,e_{N}^{k}\right)}^{T}$ for k=1,2,...,n. Since $\sum_{i=1}^{N}e_{i}^{T}\left(t\right)\mathrm{sign}\left(e_{i}\left(t\right)\right){\left|e_{i}\left(t\right)\right|}^{\mu }=$$\sum_{i=1}^{N}|e_{i}^{T}\left(t\right)||e_{i}{\left(t\right)|}^{\mu }=\sum_{i=1}^{N}\sum_{k=1}^{n}{\left|e_{i}^{k}\left(t\right)\right|}^{1+\mu }$ and by Lemma 1,

$${\left(\sum_{i=1}^{N}\sum_{k=1}^{n}{\left|e_{i}^{k}\left(t\right)\right|}^{1+\mu }\right)}^{\frac{1}{1+\mu }}\geq {\left(\sum_{i=1}^{N}\sum_{k=1}^{n}{\left|e_{i}^{k}\left(t\right)\right|}^{2}\right)}^{\frac{1}{2}}.$$

Hence,

$$\sum_{i=1}^{N}\sum_{k=1}^{n}{\left|e_{i}^{k}\left(t\right)\right|}^{1+\mu }\geq {\left(\sum_{i=1}^{N}\sum_{k=1}^{n}{\left|e_{i}^{k}\left(t\right)\right|}^{2}\right)}^{\frac{1+\mu }{2}}={\left(\sum_{i=1}^{N}e_{i}^{T}\left(t\right)e_{i}\left(t\right)\right)}^{\frac{1+\mu }{2}}.$$

Similarly,

$$\sum_{i=1}^{N}\sum_{k=1}^{n}{\left|e_{i}^{k}\left(t\right)\right|}^{1+\delta }\geq {\left(nN\right)}^{-\delta }{\left(\sum_{i=1}^{N}\sum_{k=1}^{n}{\left|e_{i}^{k}\left(t\right)\right|}^{2}\right)}^{\frac{1+\delta }{2}}={\left(nN\right)}^{-\delta }{\left(\sum_{i=1}^{N}e_{i}^{T}\left(t\right)e_{i}\left(t\right)\right)}^{\frac{1+\delta }{2}}.$$

Then, combining with (9), we can obtain

(10) $$\begin{array}{ccc} \dot{V}\left(t\right) & \leq & p_{1}V\left(t\right)-2\eta_{3}V^{\frac{1+\mu }{2}}\left(t\right)-2\eta_{4}{\left(nN\right)}^{-\delta }V^{\frac{1+\delta }{2}}\left(t\right), \end{array}$$

where $p_{1}=\lambda_{\max}\left(\left(2l_{1}-2\eta_{1}-\eta_{2}\varsigma \right)I_{N}+2c\gamma_{k}\frac{B^{T}+B}{2}\right)$.

Hence, from Lemma 3, the network (5) is fixed-time synchronized with the time $T_{1}^{*}$. □

### 3.2. Adaptive State Control

To obtain the fixed-time synchronization, we design the following adaptive control scheme.

(11) $$u_{i}\left(t\right)=\begin{array}{c} -\eta_{i}^{1}\left(t\right)e_{i}\left(t\right)-\eta_{2}e_{i}\left(t-\tau \left(t\right)\right)-\eta_{4}\mathrm{sign}\left(e_{i}\left(t\right)\right){\left|e_{i}\left(t\right)\right|}^{\delta }, \end{array}$$

where $\eta_{2},\;\eta_{4}>0$ are the control strengths, the real number δ satisfies δ>1, and the adaptive update law is given by

(12) $$\begin{array}{ccc} {\dot{\eta }}_{i}^{1}\left(t\right) & = & \varepsilon_{1}e_{i}^{T}\left(t\right)e_{i}\left(t\right)-\varepsilon_{2}\mathrm{sign}\left(\eta_{i}^{1}\left(t\right)-{\tilde{\eta }}_{1}\right)-\varepsilon_{3}\mathrm{sign}\left(\eta_{i}^{1}\left(t\right)-{\tilde{\eta }}_{1}\right){\left|\eta_{i}^{1}\left(t\right)-{\tilde{\eta }}_{1}\right|}^{\delta }, \end{array}$$

where $\varepsilon_{1},\;\varepsilon_{2},\;\varepsilon_{3}>0,$${\tilde{\eta }}_{1}$ is a constant to be determined.

According to the control law (12), the error dynamical system is then governed as follows:

(13) $$\begin{array}{ccc} {\dot{e}}_{i}\left(t\right) & = & \tilde{f}\left(t,x_{i},\Pi ,x_{i}^{\tau },\Pi^{\tau }\right)+c\sum_{j=1}^{N}b_{ij}\Gamma e_{j}\left(t\right)-\eta_{i}^{1}\left(t\right)e_{i}\left(t\right)-\eta_{2}e_{i}\left(t-\tau \left(t\right)\right)\\ \; & \; & -\eta_{4}\mathrm{sign}\left(e_{i}\left(t\right)\right){\left|e_{i}\left(t\right)\right|}^{\delta }, \end{array}$$

where $\tilde{f}\left(t,x_{i},\Pi ,x_{i}^{\tau },\Pi^{\tau }\right)=f\left(t,x_{i}\left(t\right),x_{i}\left(t-\tau \left(t\right)\right)\right)-f\left(t,\Pi (t),\Pi (t-\tau (t))\right)$.

**Theorem** **2.** *Under Assumption 1 and the controller (11), if*

$$2l_{2}-\frac{\eta_{2}}{\varsigma }\leq 0,\lambda_{\max}\left(\left(2l_{1}-2{\tilde{\eta }}_{1}-\eta_{2}\varsigma +1\right)I_{N}+2c\gamma_{k}\frac{B^{T}+B}{2}\right)<0,\;$$

*where $p_{2}=\min\left\{1,\;\frac{2\varepsilon_{2}}{\sqrt{\varepsilon_{1}}}\right\},\;\lambda_{1}=\min\left\{2\eta_{4},\;2\varepsilon_{3}{\left(\varepsilon_{1}\right)}^{\frac{\delta -1}{2}}\right\},$ then, the controlled delayed dynamical network (5) is the fixed-time synchronized. Moreover, the synchronized settling time is estimated by*

$$T\leq T_{2}^{*}=\frac{1}{p_{2}\left(1-\frac{1}{2}\right)}+\frac{1}{\lambda_{1}{\left(\left(n+1\right)N\right)}^{-\delta }\left(\frac{1+\delta }{2}-1\right)}.$$

**Proof.**  Construct the Lyapunov function as

(14) $$\begin{array}{ccc} V(t) & = & \sum_{i=1}^{N}e_{i}^{T}\left(t\right)e_{i}\left(t\right)+\sum_{i=1}^{N}\frac{1}{\varepsilon_{1}}{\left(\eta_{i}^{1}\left(t\right)-{\tilde{\eta }}_{1}\right)}^{2}. \end{array}$$

Then, its derivative along with solutions of (13) can be given as below.

(15) $$\begin{array}{ccc} \dot{V}\left(t\right) & = & 2\sum_{i=1}^{N}e_{i}^{T}\left(t\right)[\tilde{f}\left(t,x_{i},\Pi ,x_{i}^{\tau },\Pi^{\tau }\right)+c\sum_{j=1}^{N}b_{ij}\Gamma e_{j}\left(t\right)-\eta_{i}^{1}\left(t\right)e_{i}\left(t\right)\\ & \; & -\eta_{2}\left(t\right)e_{i}\left(t-\tau \left(t\right)\right)-\eta_{4}\mathrm{sign}\left(e_{i}\left(t\right)\right){\left|e_{i}\left(t\right)\right|}^{\delta }]\\ & \; & +2\sum_{i=1}^{N}\frac{1}{\varepsilon_{1}}\left(\eta_{i}^{1}\left(t\right)-{\tilde{\eta }}_{1}\right)[\varepsilon_{1}e_{i}^{T}\left(t\right)e_{i}\left(t\right)-\varepsilon_{2}\mathrm{sign}\left(\eta_{i}^{1}\left(t\right)-{\tilde{\eta }}_{1}\right)\\ & \; & -\varepsilon_{3}\mathrm{sign}\left(\eta_{i}^{1}\left(t\right)-{\tilde{\eta }}_{1}\right){\left|\eta_{i}^{1}\left(t\right)-{\tilde{\eta }}_{1}\right|}^{\delta }]\\ & \leq & 2l_{1}\sum_{i=1}^{N}e_{i}^{T}\left(t\right)e_{i}\left(t\right)+2l_{2}\sum_{i=1}^{N}e_{i}^{T}\left(t-\tau \left(t\right)\right)e_{i}\left(t-\tau \left(t\right)\right)+2c\sum_{i=1}^{N}\sum_{j=1}^{N}e_{i}^{T}\left(t\right)\\ & \; & \times b_{ij}\Gamma e_{j}\left(t\right)-2\eta_{i}^{1}\left(t\right)\sum_{i=1}^{N}e_{i}^{T}\left(t\right)e_{i}\left(t\right)-2\eta_{2}\sum_{i=1}^{N}e_{i}^{T}\left(t\right)e_{i}\left(t-\tau \left(t\right)\right)\\ & \; & -2\eta_{4}\sum_{i=1}^{N}e_{i}^{T}\left(t\right)\mathrm{sign}\left(e_{i}\left(t\right)\right){\left|e_{i}\left(t\right)\right|}^{\delta }+2\sum_{i=1}^{N}\left(\eta_{i}^{1}\left(t\right)-{\tilde{\eta }}_{1}\right)e_{i}^{T}\left(t\right)e_{i}\left(t\right)\\ & \; & -2\sum_{i=1}^{N}\frac{\varepsilon_{2}}{\varepsilon_{1}}|\eta_{i}^{1}\left(t\right)-{\tilde{\eta }}_{1}|-2\sum_{i=1}^{N}\frac{\varepsilon_{3}}{\varepsilon_{1}}{\left|\eta_{i}^{1}\left(t\right)-{\tilde{\eta }}_{1}\right|}^{\delta +1}\\ \; & \leq & \sum_{i=1}^{N}\sum_{k=1}^{n}e_{i}^{k}\left(t\right)\left(2l_{1}-\eta_{2}\varsigma -2{\tilde{\eta }}_{1}\right)e_{i}^{k}\left(t\right)+2c\sum_{i=1}^{N}\sum_{j=1}^{N}\sum_{k=1}^{n}\gamma_{k}e_{i}^{k}\left(t\right)\\ & \; & \times \frac{b_{ji}+b_{ij}}{2}e_{j}^{k}\left(t\right)+\left(2l_{2}-\frac{\eta_{2}}{\varsigma }\right)\sum_{i=1}^{N}e_{i}^{T}\left(t-\tau \left(t\right)\right)e_{i}\left(t-\tau \left(t\right)\right)\\ & \; & -2\eta_{4}\sum_{i=1}^{N}e_{i}^{T}\left(t\right)\mathrm{sign}\left(e_{i}\left(t\right)\right){\left|e_{i}\left(t\right)\right|}^{\delta }\\ & \; & -2\sum_{i=1}^{N}\frac{\varepsilon_{2}}{\varepsilon_{1}}|\eta_{i}^{1}\left(t\right)-{\tilde{\eta }}_{1}|-2\sum_{i=1}^{N}\frac{\varepsilon_{3}}{\varepsilon_{1}}{\left|\eta_{i}^{1}\left(t\right)-{\tilde{\eta }}_{1}\right|}^{\delta +1} \end{array}$$

$$\begin{array}{ccc} & \leq & \sum_{k=1}^{n}{\left({\tilde{e}}^{k}\left(t\right)\right)}^{T}\left[\left(2l_{1}-\eta_{2}\varsigma -2{\tilde{\eta }}_{1}+1\right)I_{N}+2c\gamma_{k}\frac{B^{T}+B}{2}\right]{\tilde{e}}^{k}\left(t\right)\\ & & -\sum_{i=1}^{N}\sum_{k=1}^{n}|e_{i}^{k}\left(t\right)|-2\eta_{4}\sum_{i=1}^{N}e_{i}^{T}\left(t\right)\mathrm{sign}\left(e_{i}\left(t\right)\right){\left|e_{i}\left(t\right)\right|}^{\delta }\\ & & -2\sum_{i=1}^{N}\frac{\varepsilon_{2}}{\varepsilon_{1}}|\eta_{i}^{1}\left(t\right)-{\tilde{\eta }}_{1}|-2\sum_{i=1}^{N}\frac{\varepsilon_{3}}{\varepsilon_{1}}{\left|\eta_{i}^{1}\left(t\right)-{\tilde{\eta }}_{1}\right|}^{\delta +1},\\ & \leq & -\sum_{i=1}^{N}\sum_{k=1}^{n}|e_{i}^{k}\left(t\right)|-2\eta_{4}\sum_{i=1}^{N}e_{i}^{T}\left(t\right)\mathrm{sign}\left(e_{i}\left(t\right)\right){\left|e_{i}\left(t\right)\right|}^{\delta }\\ & & -2\sum_{i=1}^{N}\frac{\varepsilon_{2}}{\varepsilon_{1}}|\eta_{i}^{1}\left(t\right)-{\tilde{\eta }}_{1}|-2\sum_{i=1}^{N}\frac{\varepsilon_{3}}{\varepsilon_{1}}{\left|\eta_{i}^{1}\left(t\right)-{\tilde{\eta }}_{1}\right|}^{\delta +1}\\ & \leq & -{\left[\sum_{i=1}^{N}\sum_{k=1}^{n}|e_{i}^{k}{\left(t\right)|}^{2}+4\sum_{i=1}^{N}\frac{{\left(\varepsilon_{2}\right)}^{2}}{{\left(\varepsilon_{1}\right)}^{2}}{\left|\eta_{i}^{1}\left(t\right)-{\tilde{\eta }}_{1}\right|}^{2}\right]}^{\frac{1}{2}}\\ & & -2\eta_{4}\sum_{i=1}^{N}e_{i}^{T}\left(t\right)\mathrm{sign}\left(e_{i}\left(t\right)\right)|e_{i}{\left(t\right)|}^{\delta }-2\sum_{i=1}^{N}\frac{\varepsilon_{3}}{\varepsilon_{1}}{\left|\eta_{i}^{1}\left(t\right)-{\tilde{\eta }}_{1}\right|}^{\delta +1}. \end{array}$$

Since $\sum_{i=1}^{N}e_{i}^{T}\left(t\right)\mathrm{sign}\left(e_{i}\left(t\right)\right)|e_{i}{\left(t\right)|}^{\delta }=\sum_{i=1}^{N}|e_{i}^{T}\left(t\right)||e_{i}{\left(t\right)|}^{\delta }=\sum_{i=1}^{N}\sum_{k=1}^{n}{\left|e_{i}^{k}\left(t\right)\right|}^{1+\delta }$, hence,

$$\begin{array}{c} 2\eta_{4}\sum_{i=1}^{N}e_{i}^{T}\left(t\right)\mathrm{sign}\left(e_{i}\left(t\right)\right)|e_{i}{\left(t\right)|}^{\delta }+2\sum_{i=1}^{N}\frac{\varepsilon_{4}}{\varepsilon_{1}}{\left|\eta_{i}^{1}\left(t\right)-{\tilde{\eta }}_{1}\right|}^{1+\delta }\\ =2\eta_{4}\sum_{i=1}^{N}\sum_{k=1}^{n}{\left|e_{i}^{k}\left(t\right)\right|}^{1+\delta }+2\varepsilon_{3}{\left(\varepsilon_{1}\right)}^{\frac{\delta -1}{2}}\sum_{i=1}^{N}{\left(\frac{1}{\sqrt{\varepsilon_{1}}}\left|\eta_{i}^{1}\left(t\right)-{\tilde{\eta }}_{1}\right|\right)}^{1+\delta }\\ \geq \lambda_{1}\left[\sum_{i=1}^{N}\sum_{k=1}^{n}{\left|e_{i}^{k}\left(t\right)\right|}^{1+\delta }+\sum_{i=1}^{N}{\left(\frac{1}{\sqrt{\varepsilon_{1}}}\left|\eta_{i}^{1}\left(t\right)-{\tilde{\eta }}_{1}\right|\right)}^{1+\delta }\right]\\ \geq \lambda_{1}{\left(\left(n+1\right)N\right)}^{-\delta }V^{\frac{1+\delta }{2}}\left(t\right), \end{array}$$

where $\lambda_{1}=\min\left\{2\eta_{4},\;2\varepsilon_{3}{\left(\varepsilon_{1}\right)}^{\frac{\delta -1}{2}}\right\}$. Then, combining with (15), we can obtain

(16) $$\begin{array}{ccc} \dot{V}\left(t\right) & \leq & -p_{2}V^{\frac{1}{2}}\left(t\right)-\lambda_{1}{\left(\left(n+1\right)N\right)}^{-\delta }V^{\frac{1+\delta }{2}}\left(t\right), \end{array}$$

where $p_{2}=\min\left\{1,\;\frac{2\varepsilon_{2}}{\sqrt{\varepsilon_{1}}}\right\}.$

Hence, the network (5) is fixed-time synchronized with the time $T_{2}^{*}$. □

As a special case, a type of coupled delayed neural network is considered.

(17) $$\begin{array}{ccc} {\dot{x}}_{i}\left(t\right) & = & -Ax_{i}\left(t\right)+\mathrm{Dg}\left(x_{i}\left(t\right)\right)+\mathrm{Cg}\left(x_{i}\left(t-\tau \left(t\right)\right)\right)+c\sum_{j=1}^{N}b_{ij}\Gamma x_{j}\left(t\right)+u_{i}\left(t\right), \end{array}$$

where $i\in I,\;b_{ij}$ is defined in (2), $x_{i}\left(t\right)={\left(x_{i}^{1}\left(t\right),x_{i}^{2}\left(t\right)...,x_{i}^{n}\left(t\right)\right)}^{T}\in R^{n}$ denotes the state variable associated with the *i*th neuron, $A=\mathrm{diag}\{a_{1},a_{2},...,a_{n}\}$ is the decay constant matrix with $a_{i}>0$ for i=1,2,...,n, $D={\left(d_{ij}\right)}_{n\times n}$ and $C={\left(c_{ij}\right)}_{n\times n}$ are the connection matrix and delayed connection matrix, respectively, and $g\left(x_{i}\left(t\right)\right)={\left(g_{1}\left(x_{i}^{1}\left(t\right)\right),g_{2}\left(x_{i}^{2}\left(t\right)\right),...,g_{n}\left(x_{i}^{n}\left(t\right)\right)\right)}^{T}$ is the activation function of the neurons and satisfies the following condition.

**Assumption** **2.** *There exists a positive constant s>0 such that*

$${\left(g(x)-g(y)\right)}^{T}\left(g(x)-g(y)\right)\leq s{\left(x-y\right)}^{T}\left(x-y\right),$$

*for any $x,y\in R^{n}$.*

Correspondingly, the synchronization equation Π(t) of (17) is represented by

(18) $$\begin{array}{ccc} \dot{\Pi }\left(t\right) & = & -A\Pi (t)+Dg(\Pi (t))+Cg(\Pi (t-\tau (t))). \end{array}$$

**Theorem** **3.** *Under Assumption 2 and the controller (6), if*

$$p_{1}<\min\left\{2\eta_{3},\;2\eta_{4}{\left(nN\right)}^{-\delta }\right\},\;s-\frac{\eta_{2}}{\varsigma }\leq 0,$$

*where $p_{1}=\lambda_{\max}\left(\left(\lambda -2\eta_{1}-\eta_{2}\varsigma \right)I_{N}+2c\gamma_{k}\frac{B^{T}+B}{2}\right),\;\lambda =\lambda_{\max}\left(-A-A^{T}+{\mathrm{DD}}^{T}+{\mathrm{CC}}^{T}+sI_{N}\right)$, then, the controlled delayed dynamical network (17) is the fixed-time synchronized. Moreover, the synchronized settling time is estimated by*

$$T\leq T_{1}^{*}=\frac{1}{p_{1}\left(1-\frac{1+\mu }{2}\right)}\ln\left(\frac{2\eta_{3}}{2\eta_{3}-p_{1}}\right)+\frac{1}{p_{1}\left(\frac{1+\delta }{2}-1\right)}\ln\left(\frac{2\eta_{4}{\left(nN\right)}^{-\delta }}{2\eta_{4}{\left(nN\right)}^{-\delta }-p_{1}}\right).$$

**Proof.**  Under Assumption 1, the network is fixed-time synchronized from Theorem 1. Using Lemma 2 and Assumption 2, we can get

$$\begin{array}{c} \;\;\;\;{\left(x_{i}\left(t\right)-y_{i}\left(t\right)\right)}^{T}\left(f\left(t,x_{i}\left(t\right),x_{i}\left(t-\tau \left(t\right)\right)\right)-f\left(t,y_{i}\left(t\right),y_{i}\left(t-\tau \left(t\right)\right)\right)\right)\\ ={\left(x_{i}\left(t\right)-y_{i}\left(t\right)\right)}^{T}[-A\left(x_{i}\left(t\right)-y_{i}\left(t\right)\right)+D\left(g\left(x_{i}\left(t\right)\right)-g\left(y_{i}\left(t\right)\right)\right)\\ \;\;\;+C(g\left(x_{i}\left(t-\tau \left(t\right)\right)\right)-g\left(y_{i}\left(t-\tau \left(t\right)\right)\right))]\\ \leq \frac{1}{2}{\left(x_{i}\left(t\right)-y_{i}\left(t\right)\right)}^{T}\left(-A-A^{T}+{\mathrm{DD}}^{T}+{\mathrm{CC}}^{T}+sI_{N}\right)\left(x_{i}\left(t\right)-y_{i}\left(t\right)\right)\\ \;\;\;+\frac{s}{2}{\left(x_{i}\left(t-\tau \left(t\right)\right)-y_{i}\left(t-\tau \left(t\right)\right))\right)}^{T}\left(x_{i}\left(t-\tau \left(t\right)\right)-y_{i}\left(t-\tau \left(t\right)\right))\right)\\ \leq \frac{\lambda }{2}{\left(x_{i}\left(t\right)-y_{i}\left(t\right)\right)}^{T}\left(x_{i}\left(t\right)-y_{i}\left(t\right)\right)+\frac{s}{2}{\left(x_{i}\left(t-\tau \left(t\right)\right)-y_{i}\left(t-\tau \left(t\right)\right))\right)}^{T}\\ \;\;\;\times \left(x_{i}\left(t-\tau \left(t\right)\right)-y_{i}\left(t-\tau \left(t\right)\right))\right), \end{array}$$

where $\lambda =\lambda_{\max}\left(-A-A^{T}+{\mathrm{DD}}^{T}+{\mathrm{CC}}^{T}+sI_{N}\right)$, which shows that assumption 1 holds, and $l_{1}=\frac{\lambda }{2}$, $l_{2}=\frac{s}{2}$. Hence, from Theorem 1, the coupled network (17) is fixed-time synchronized to (18). The proof of Theorem 3 is completed. □

**Theorem** **4.** *Under Assumption 2 and the controller (11), if*

$$s-\frac{\eta_{2}}{\varsigma }\leq 0,\lambda_{\max}\left(\left(\lambda -2{\tilde{\eta }}_{1}-\eta_{2}\varsigma +1\right)I_{N}+2c\gamma_{k}\frac{B^{T}+B}{2}\right)<0,\;$$

*where $p_{2}=\min\left\{1,\;\frac{2\varepsilon_{2}}{\sqrt{\varepsilon_{1}}}\right\},\;\lambda_{1}=\min\left\{2\eta_{4},\;\varepsilon_{3}{\left(\varepsilon_{1}\right)}^{\frac{\delta -1}{2}}\right\},\;\lambda =\lambda_{\max}\left(-A-A^{T}+{\mathrm{DD}}^{T}+{\mathrm{CC}}^{T}+sI_{N}\right),$ then, the dynamical network (17) is the fixed-time synchronized. Moreover, the synchronized settling time is estimated by*

$$T\leq T_{2}^{*}=\frac{1}{p_{2}\left(1-\frac{1}{2}\right)}+\frac{1}{\lambda_{1}{\left(\left(n+1\right)N\right)}^{-\delta }\left(\frac{1+\delta }{2}-1\right)}.$$

Suppose τ(t)=0, network model (5) can be rewritten as follows.

(19) $$\begin{array}{ccc} {\dot{x}}_{i}\left(t\right) & = & f\left(t,x_{i}\left(t\right)\right)+c\sum_{j=1}^{N}b_{ij}\Gamma x_{j}\left(t\right)+u_{i}\left(t\right). \end{array}$$

The feedback control gain $u_{i}\left(t\right)$ in Equation (6) becomes the following form.

(20) $$u_{i}\left(t\right)=\begin{array}{c} -\eta_{1}e_{i}\left(t\right)-\eta_{3}\mathrm{sign}\left(e_{i}\left(t\right)\right)|e_{i}{\left(t\right)|}^{\mu }-\eta_{4}\mathrm{sign}\left(e_{i}\left(t\right)\right){\left|e_{i}\left(t\right)\right|}^{\delta }, \end{array}$$

where $\eta_{1},\;\eta_{3},\;\eta_{4}>0$ are the control gains, and μ satisfies 0≤μ<1, δ satisfies δ>1.

Moreover, the adaptive control gain $u_{i}\left(t\right)$ in Equation (11) becomes the following form.

(21) $$u_{i}\left(t\right)=\begin{array}{c} -\eta_{i}^{1}\left(t\right)e_{i}\left(t\right)-\eta_{4}\mathrm{sign}\left(e_{i}\left(t\right)\right){\left|e_{i}\left(t\right)\right|}^{\delta }, \end{array}$$

where $\eta_{4}>0$ is the control gain, δ satisfies δ>1, and the adaptive update law is given by

$$\begin{array}{ccc} {\dot{\eta }}_{i}^{1}\left(t\right) & = & \varepsilon_{1}e_{i}^{T}\left(t\right)e_{i}\left(t\right)-\varepsilon_{2}\mathrm{sign}\left(\eta_{i}^{1}\left(t\right)-{\tilde{\eta }}_{1}\right)-\varepsilon_{3}\mathrm{sign}\left(\eta_{i}^{1}\left(t\right)-{\tilde{\eta }}_{1}\right){\left|\eta_{i}^{1}\left(t\right)-{\tilde{\eta }}_{1}\right|}^{\delta }, \end{array}$$

where $\varepsilon_{1},\;\varepsilon_{2},\;\varepsilon_{3}>0,$${\tilde{\eta }}_{1}$ is constant to be determined.

Correspondingly, the synchronization equation associated with (19) is depicted by

(22) $$\begin{array}{ccc} \dot{\Pi }\left(t\right) & = & f(t,\Pi (t)),\;\;\;\;i\in I. \end{array}$$

Based on Theorems 1–4, we can get the following Corollaries 1–4, respectively.

**Corollary** **1.** *Under Assumption 1 and the controller (20), if*

$$p_{1}<\min\left\{2\eta_{3},\;2\eta_{4}{\left(nN\right)}^{-\delta }\right\},$$

*where $p_{1}=\lambda_{\max}\left(\left(2l_{1}-2\eta_{1}\right)I_{N}+2c\gamma_{k}\frac{B^{T}+B}{2}\right),$ then, the dynamical network (19) is the fixed-time synchronized. Moreover, the synchronized settling time is estimated by*

$$T\leq T_{1}^{*}=\frac{1}{p_{1}\left(1-\frac{1+\mu }{2}\right)}\ln\left(\frac{2\eta_{3}}{2\eta_{3}-p_{1}}\right)+\frac{1}{p_{1}\left(\frac{1+\delta }{2}-1\right)}\ln\left(\frac{2\eta_{4}{\left(nN\right)}^{-\delta }}{2\eta_{4}{\left(nN\right)}^{-\delta }-p_{1}}\right).$$

**Corollary** **2.** *Under Assumption 1 and the controller (21), if*

$$\lambda_{\max}\left(\left(2l_{1}-2{\tilde{\eta }}_{1}+1\right)I_{N}+2c\gamma_{k}\frac{B^{T}+B}{2}\right)<0,\;$$

*where $p_{2}=\min\left\{1,\;\frac{2\varepsilon_{2}}{\sqrt{\varepsilon_{1}}}\right\},\;\lambda_{1}=\min\left\{2\eta_{4},\;\varepsilon_{3}{\left(\varepsilon_{1}\right)}^{\frac{\delta -1}{2}}\right\},$ then, the dynamical network (19) is the fixed-time synchronized. Moreover, the synchronized settling time is estimated by*

$$T\leq T_{2}^{*}=\frac{1}{p_{2}\left(1-\frac{1}{2}\right)}+\frac{1}{\lambda_{1}{\left(\left(n+1\right)N\right)}^{-\delta }\left(\frac{1+\delta }{2}-1\right)}.$$

**Corollary** **3.** *Under Assumption 2 and the controller (20), if*

$$p_{1}<\min\{2\eta_{3},\;2\eta_{4}\left({\left(nN\right)}^{-\delta }\right\},$$

*where $p_{1}=\lambda_{\max}\left(\left(\lambda -2\eta_{1}\right)I_{N}+2c\gamma_{k}\frac{B^{T}+B}{2}\right),\;\lambda =\lambda_{\max}\left(-A-A^{T}+{\mathrm{DD}}^{T}+{\mathrm{CC}}^{T}+sI_{N}\right),$ then, the dynamical network (19) is the fixed-time synchronized. Moreover, the synchronized settling time is estimated by*

$$T\leq T_{1}^{*}=\frac{1}{p_{1}\left(1-\frac{1+\mu }{2}\right)}\ln\left(\frac{2\eta_{3}}{2\eta_{3}-p_{1}}\right)+\frac{1}{p_{1}\left(\frac{1+\delta }{2}-1\right)}\ln\left(\frac{2\eta_{4}{\left(nN\right)}^{-\delta }}{2\eta_{4}{\left(nN\right)}^{-\delta }-p_{1}}\right).$$

**Corollary** **4.** *Under Assumption 2 and the controller (21), if*

$$\lambda_{\max}\left(\left(\lambda -2{\tilde{\eta }}_{1}+1\right)I_{N}+2c\gamma_{k}\frac{B^{T}+B}{2}\right)<0,\;$$

*where $p_{2}=\min\left\{1,\;\frac{2\varepsilon_{2}}{\sqrt{\varepsilon_{1}}}\right\},\;\lambda_{1}=\min\left\{2\eta_{4},\;\varepsilon_{3}{\left(\varepsilon_{1}\right)}^{\frac{\delta -1}{2}}\right\},\;\lambda =\lambda_{\max}\left(-A-A^{T}+{\mathrm{DD}}^{T}+{\mathrm{CC}}^{T}+sI_{N}\right),$ then, the dynamical network (19) is the fixed-time synchronized. Moreover, the synchronized settling time is estimated by*

$$T\leq T_{2}^{*}=\frac{1}{p_{2}\left(1-\frac{1}{2}\right)}+\frac{1}{\lambda_{1}{\left(\left(n+1\right)N\right)}^{-\delta }\left(\frac{1+\delta }{2}-1\right)}.$$

**Remark** **2.** *As we know, in the process of information transmission and spreading, the communication delay is a typical phenomenon and may result in oscillation and instability dynamic behaviors. Hence, it is necessary to study the influence of time delay on network’ dynamic behavior. Otherwise, time delays were not considered in other works [14,18,19,27]. In this paper, by establishing Lyapunov function, the synchronization of delayed dynamical networks has been realized. When τ(t)=0, Corollary 3 in this paper is equivalent to Theorem 1 in the works of Li [18] and Ji [19]. In other words, results in the papers of Li [18] and Ji [19] are the special case of our results. Moreover, for τ(t), we do not require that its derivative is bounded. Hence, the model we considered in this paper is more general.*

**Remark** **3.** *In recent work [32], fixed-time synchronization of dynamic system via improving fixed-time stability was studied. However, the upper bound of synchronization time is, respectively, estimated for cases a<0 and a>0 and two different formulas of the estimate were obtained, which inevitably results in some inconvenience in applications. Different from the result of reference [32], a unified form of the estimate is derived for the cases a<0 and a>0 in Lemma 3, and it is more convenient in practice.*

**Remark** **4.** *Using Lemma 3, the network can realize fixed-time synchronization under linear feedback control and adaptive control in this paper. Feedback control and adaptive control are continuous control approaches. For continuous control approaches, such as intermittent control and impulse control, a lot of results have been obtained. However, the fixed time synchronization of networks cannot be received by using Lemma 3 under intermittent control and impulse control.*

**Remark** **5.** *Until now, for articles that have been published on fixed-time synchronization, feedback control was mainly used [19,26,27]. Unfortunately, only very few articles have considered the fixed-time synchronization through adaptive control method [18,31] to reduce the conservativeness of synchronization criteria. In this paper, both adaptive controller and feedback controller are designed to ensure the fixed-time synchronization of delayed complex networks.*

## 4. Numerical Simulations

In this section, a delayed network is provided to present fixed-time synchronization.

**Example** **1.** *Consider the coupled networks model with variable delay as follows.*

(23) $$\begin{array}{ccc} {\dot{x}}_{i}\left(t\right) & = & -Ax_{i}\left(t\right)+Dg_{1}\left(x_{i}\left(t\right)\right)+Cg_{2}\left(x_{i}\left(t-\tau \left(t\right)\right)\right)+c\sum_{j=1}^{8}b_{ij}\Gamma x_{j}\left(t\right)+u_{i}\left(t\right),\\ & \; & \;\;\;\;\;\;\;\;\;\;i=1,2,...,8, \end{array}$$

*where $x_{i}\left(t\right)=\left(x_{i}^{1}\left(t\right),x_{i}^{2}\left(t\right)\right)\in R^{2},\;i=1,2,...,8$, $g_{1}\left(u\right)=g_{2}\left(u\right)=\left(\tanh\left(u_{1}\right),\tanh\left(u_{2}\right)\right)$, c=2, and*

$$A=\left(\begin{array}{cc} 1 & 0\\ 0 & 1 \end{array}\right),\;C=\left(\begin{array}{cc} -1.5 & -0.1\\ -0.1 & -1.5 \end{array}\right),\;\Gamma =\left(\begin{array}{cc} 1 & 0\\ 0 & 1 \end{array}\right),\;D=\left(\begin{array}{cc} 2 & -0.1\\ -4 & 3 \end{array}\right),$$

$$\;B=\left(\begin{array}{cccccccc} -2 & 0.2 & 0.4 & 0.2 & 0.3 & 0.3 & 0.32 & 0.28\\ 0.72 & -4 & 0.48 & 0.48 & 0.72 & 0.56 & 0.64 & 0.4\\ 0.1 & 0.1 & -1 & 0.16 & 0.13 & 0.17 & 0.15 & 0.19\\ 0.8 & 0.6 & 0.32 & -4 & 0.64 & 0.6 & 0.64 & 0.4\\ 0.48 & 0.72 & 0.6 & 0.68 & -4 & 0.68 & 0.44 & 0.4\\ 0.2 & 0.3 & 0.3 & 0.32 & 0.2 & -2 & 0.32 & 0.36\\ 0.68 & 0.56 & 0.64 & 0.52 & 0.48 & 0.36 & -4 & 0.76\\ 0.52 & 0.72 & 0.56 & 0.64 & 0.64 & 0.52 & 0.4 & -4 \end{array}\right).$$

*In the case that network (23) reaches complete synchronization, that is, $\lim_{t\to \infty }∥x_{i}\left(t\right)-\Pi \left(t\right)∥=0,\;i=1,2,...,8$, we have the following synchronized state equation:*

(24) $$\begin{array}{ccc} \dot{\Pi }\left(t\right) & = & -A\Pi \left(t\right)+Dg_{1}\left(\Pi \left(t\right)\right)+{\mathrm{Cg}}_{2}\left(\Pi \left(t-\tau \left(t\right)\right)\right). \end{array}$$

**Figure 1:**
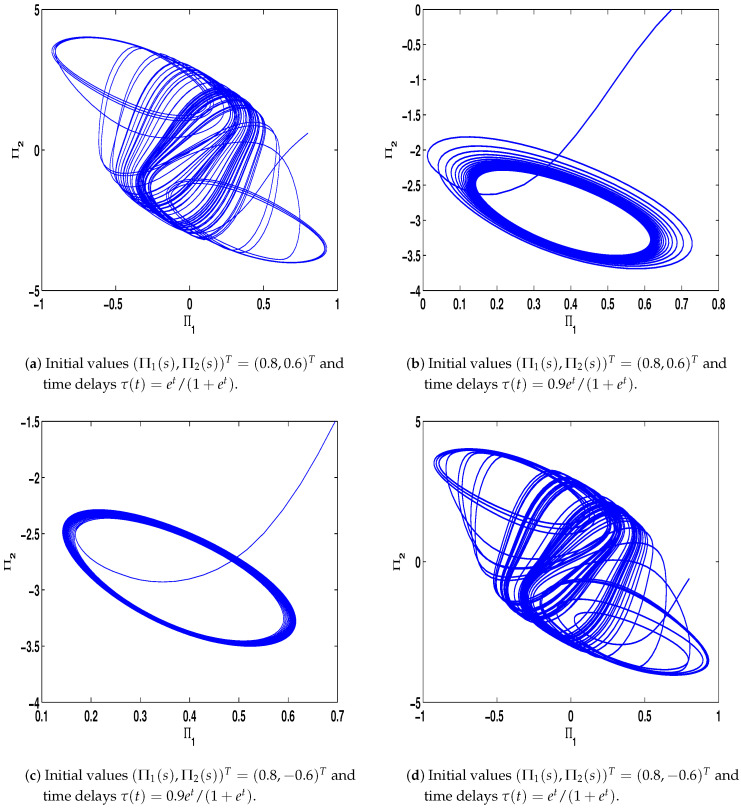
Dynamical behavior of neural networks (24) with differential initial values and differential time delays.

The dynamic property of (24) with the differential initial values ${\left(\Pi_{1}\left(s\right),\Pi_{2}\left(s\right)\right)}^{T}={\left(0.8,0.6\right)}^{T}$ or ${\left(\Pi_{1}\left(s\right),\Pi_{2}\left(s\right)\right)}^{T}={\left(0.8,-0.6\right)}^{T}$ and differential time delays $\tau \left(t\right)=e^{t}/\left(1+e^{t}\right)$ or $\tau \left(t\right)=0.9e^{t}/\left(1+e^{t}\right)$ with s∈[−1,0] can be emerged, which is revealed in Figure 1 and the states is chaotic attractor in this case. Moreover, the different dynamic properties of (23) with the initial values $x_{1}^{1}=0.1,\;x_{2}^{1}=-0.45,\;x_{3}^{1}=0.45,\;x_{4}^{1}=0.2,\;x_{5}^{1}=-0.2,\;x_{6}^{1}=1,\;x_{7}^{1}=2,\;x_{8}^{1}=-1,\;x_{1}^{2}=-2,\;x_{2}^{2}=2,\;x_{3}^{2}=-0.5,\;x_{4}^{2}=1,\;x_{5}^{2}=0.8,\;x_{6}^{2}=-0.4,\;x_{7}^{2}=0.6,\;x_{8}^{2}=0.4$ with s∈[−1,0] are given in the following.

### 4.1. Discontinuous Feedback Control

**Figure 2:**
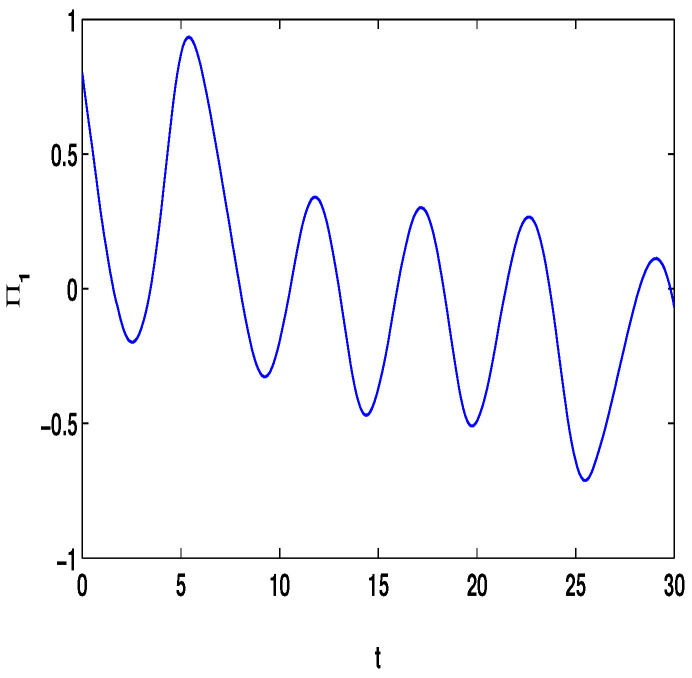
The state of $\Pi_{1}\left(t\right)$ with $(\Pi_{1}\left(s\right)$,$\Pi_{2}{\left(s\right))}^{T}=$${\left(0.8,0.6\right)}^{T}$ and $\tau \left(t\right)=e^{t}/\left(1+e^{t}\right)$.

**Figure 3:**
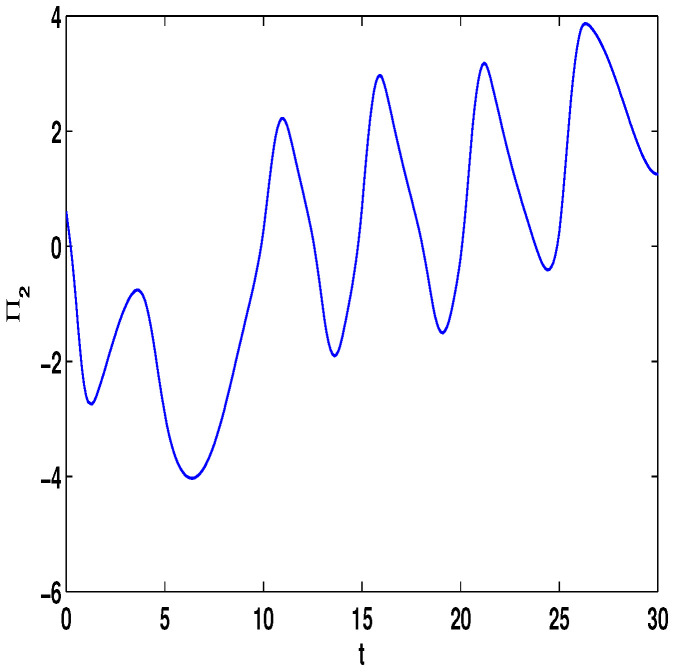
The state of $\Pi_{2}\left(t\right)$ with $(\Pi_{1}\left(s\right)$,$\Pi_{2}{\left(s\right))}^{T}=$${\left(0.8,0.6\right)}^{T}$ and $\tau \left(t\right)=e^{t}/\left(1+e^{t}\right)$.

**Figure 4:**
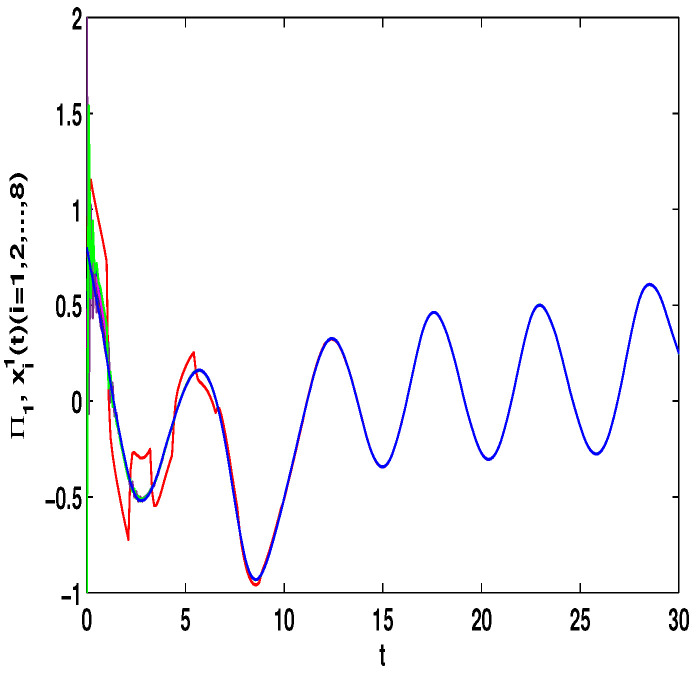
The synchronization of state $x_{i}^{1}\left(t\right)$ and $\Pi_{1}\left(t\right)$.

**Figure 5:**
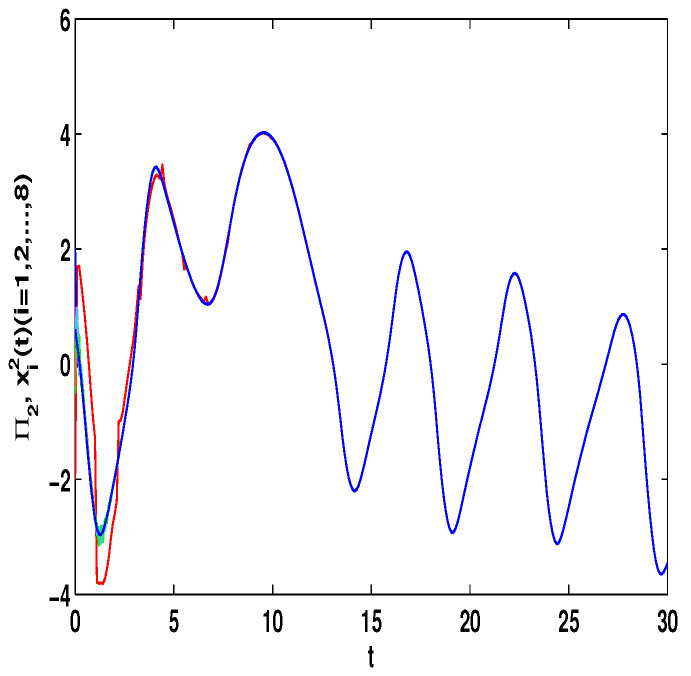
The synchronization of state $x_{i}^{2}\left(t\right)$ and $\Pi_{2}\left(t\right)$.

**Figure 6:**
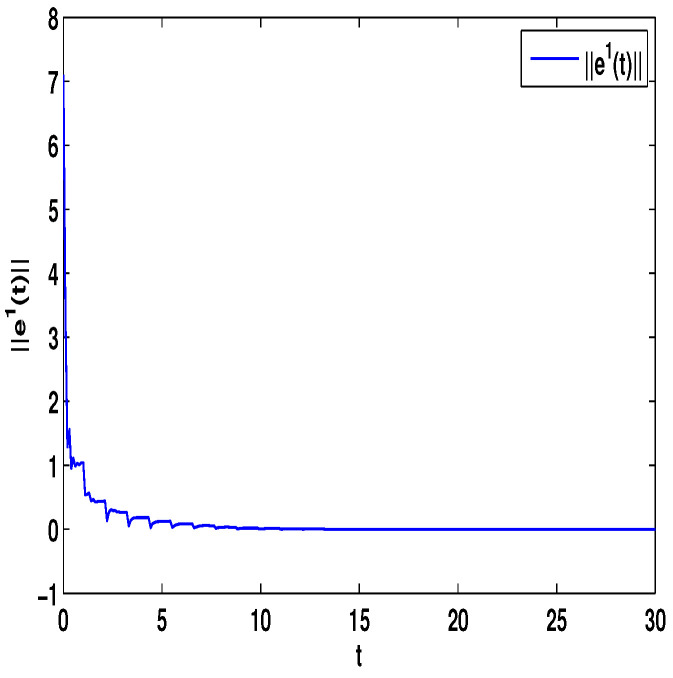
The synchronization error $\left|\right|e^{1}\left|\right|=\sum_{i=1}^{8}\left|x_{i}^{1}-\Pi_{1}\right|$.

**Figure 7:**
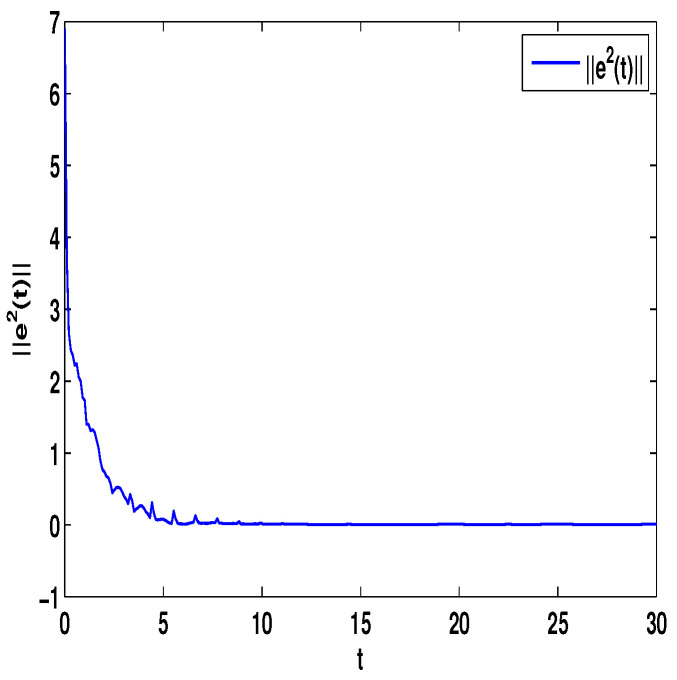
The synchronization error $\left|\right|e^{2}\left|\right|=\sum_{i=1}^{8}\left|x_{i}^{2}-\Pi_{2}\right|$.

Let $\eta_{1}=2,\;\eta_{2}=5,\;\eta_{3}=4,\;\eta_{4}=8,\;\mu =0.9,\;\delta =1.05,\;s=1,\;\varsigma =4$. By computation, $\lambda =\lambda_{\max}\left(-A-A^{T}+{\mathrm{DD}}^{T}+{\mathrm{CC}}^{T}+sI_{N}\right)=24.5047,\;p_{1}=0.5077$, then, the conditions in Theorem 3 are satisfied. From Theorem 3, the networks (23) under the controller (6) can be synchronized with fixed-time $T<T_{1}^{*}=71.65$. Figure 2 and Figure 3 show the dynamics of $\Pi_{1}$ and $\Pi_{2}$, respectively. Figure 4 and Figure 5 show the synchronization of dynamics, and Figure 6 and Figure 7 show the errors of dynamics.

### 4.2. Adaptive State Control

**Figure 8:**
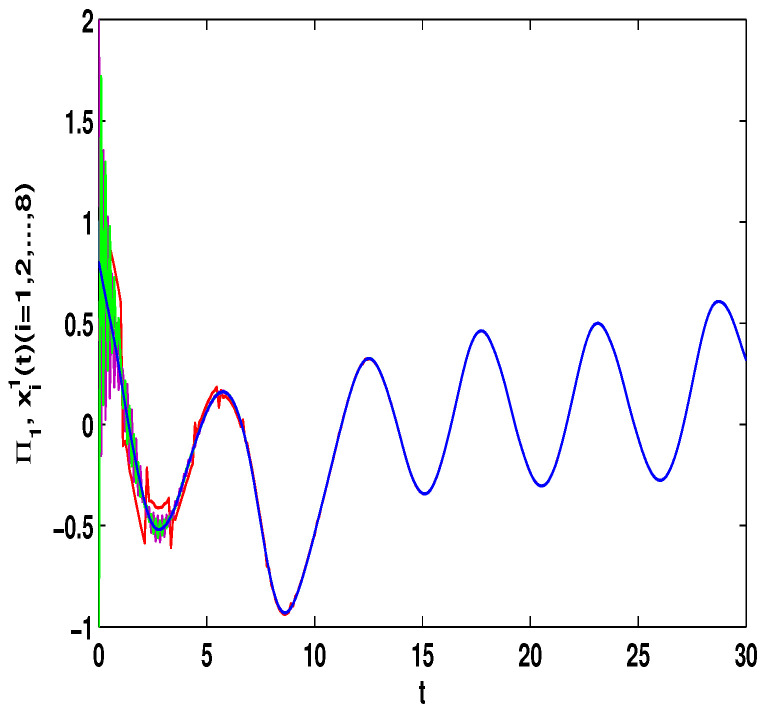
The synchronization of state $x_{i}^{1}\left(t\right)$ and $\Pi_{1}\left(t\right)$.

**Figure 9:**
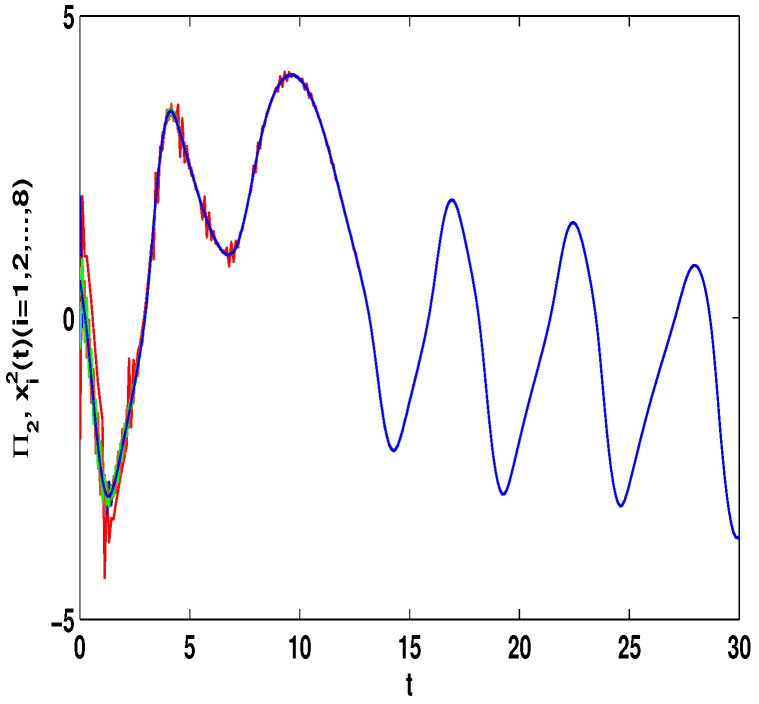
The synchronization of state $x_{i}^{2}\left(t\right)$ and $\Pi_{2}\left(t\right)$.

**Figure 10:**
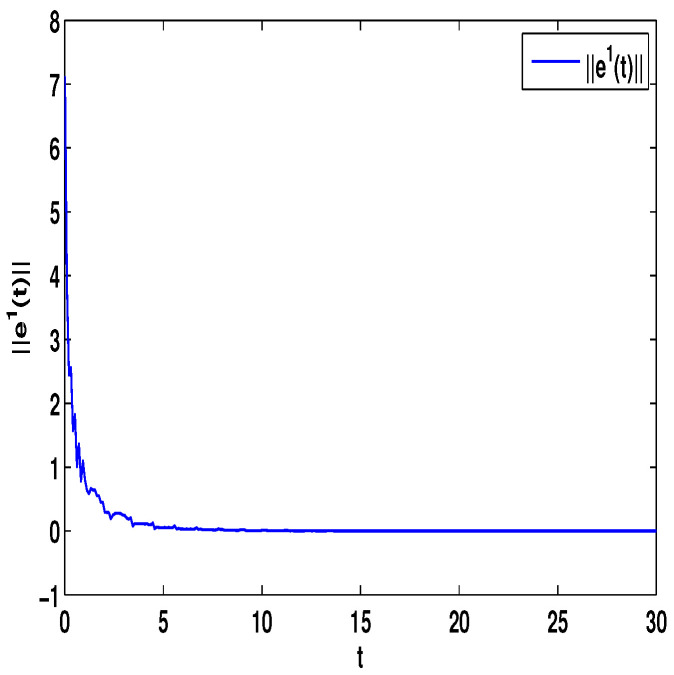
The synchronization error $\left|\right|e^{1}\left|\right|=\sum_{i=1}^{8}\left|x_{i}^{1}-\Pi_{1}\right|$.

**Figure 11:**
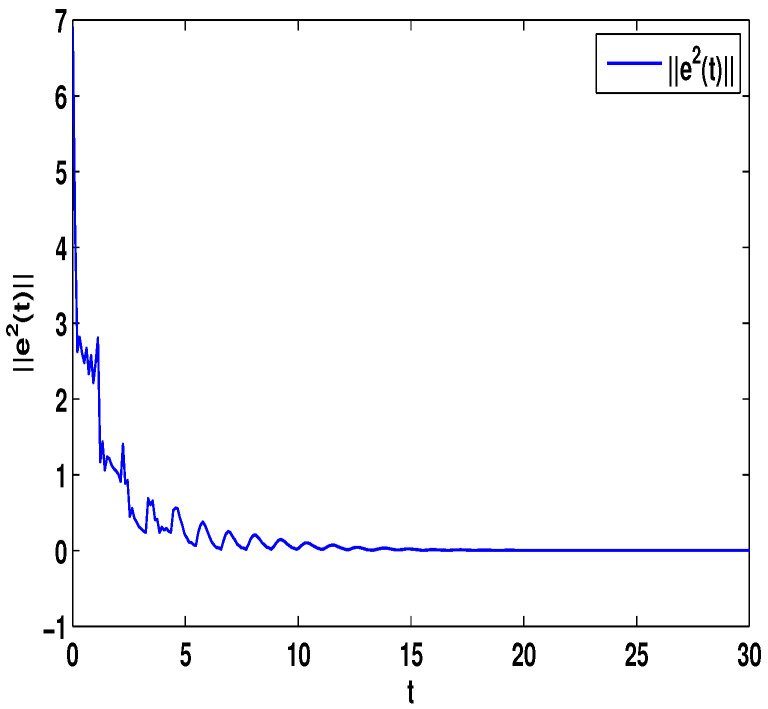
The synchronization error $\left|\right|e^{2}\left|\right|=\sum_{i=1}^{8}\left|x_{i}^{2}-\Pi_{2}\right|$.

**Figure 12:**
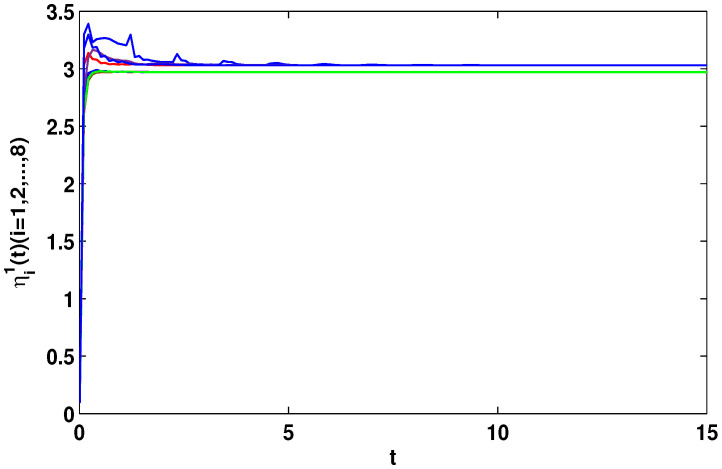
Adaptive control gain $\eta_{i}^{1}\left(t\right)$ with $\eta_{i}^{1}\left(0\right)=0.1$ for i=1,2,...,8.

Let $\eta_{2}=5,\;\eta_{4}=8,\;\mu =0.9,\;\delta =1.05,\;s=1,\;\varsigma =4$, and $\varepsilon_{1}=2,\;\varepsilon_{2}=0.2,\;\varepsilon_{3}=20,\;{\tilde{\eta }}_{1}=3$. By computation, $\lambda =\lambda_{\max}\left(-A-A^{T}+{\mathrm{DD}}^{T}+{\mathrm{CC}}^{T}+sI_{N}\right)=24.5047,\;\lambda_{\max}\left(\left(\lambda -2{\tilde{\eta }}_{1}-\eta_{2}\varsigma +1\right)I_{N}+2c\gamma_{k}\frac{B^{T}+B}{2}\right)=-0.4923<0,\;p_{2}=0.2829,\;\lambda_{1}=7.7276$, then, we can obtain the conditions of Theorem 4 are satisfied. From Theorem 4, the networks (23) under the controller (11) can be synchronized with fixed-time $T<T_{2}^{*}=62.37$. Figure 8 and Figure 9 show the synchronization of dynamics, and Figure 10 and Figure 11 show the errors of dynamics. Time evolution of adaptive control gain $\eta_{i}^{1}\left(t\right)$ with $\eta_{i}^{1}\left(0\right)=0.1$ for i=1,2,...,8 is given in Figure 12.

**Remark** **6.** *From Figure 1, the system (24) with time delay is a chaotic system. Eight nodes are chosen in the slave system and by simple computing the values of some corresponding parameters and choosing the values of $\eta_{1},\;\eta_{2},\;\eta_{3},\;\eta_{4},\;\mu ,\;\delta ,\;\varepsilon_{1},\;\varepsilon_{2},\;\varepsilon_{3},$ the conditions of Theorems 3 and 4 are satisfied. From Lemma 3, by applying the adaptive and feedback controllers to the system (23), the system (23) and the corresponding system (24) can be reached synchronization. The synchronized states of systems (23) and (24) are given in Figure 4, Figure 5, Figure 8 and Figure 9, and the errors of the systems (23) and (24) are shown in Figure 6, Figure 7, Figure 10 and Figure 11. It can be seen that the synchronization of systems (23) and (24) is indeed solved, and the simulation results demonstrate the theoretical analysis very well.*

## 5. Conclusions

In this paper, the fixed-time synchronization for a class delayed complex networks model is investigated under feedback control strategy and adaptive control strategy. Firstly, the complex network model we studied has time delay. Secondly, by constructing simple Lyapunov function, we give some ordinary yet useful sufficient criteria of delayed complex networks. Furthermore, when τ(t)=0, we get a special cases, which is considered in references [14,18,19,27,35]. In addition, for τ(t), we do not require that its derivative is bounded. In this meaning, the results obtained in this paper are more general. Finally, the numerical examples are given to show the validness of the corresponding scheme.

Generally speaking, the fixed-time synchronization settling time and its estimation of the networks depend on the initial value. However, the synchronized time in PAT synchronization can be independent of any initial value and any parameter and pre-specified according to actual needs. As we know, PAT synchronization of delayed dynamic networks are few investigated. Hence, it is meaningful to address this problem in our recent research topics.

## Data Availability

Not applicable.

## Abbreviations

The following abbreviations are used in this manuscript:

MASs	Complex networks model
FONMASs	Delayed complex networks model
SMC	Feedback control and adaptive control

## References

[1] Chua L., Roska T. (2002). Cellular Neural Networks and Visual Computing: Foundation and Applications.

[2] Zhang X., Zhou Y., Wang J., Lu X. (2021). Personal interest attention graph neural networks for session-based recommendation. Entropy.

[3] Faloutsos M., Faloutsos P., Faloutsos C. (1999). On power-law relationships of the Interact topology. Comput. Commun. Rev..

[4] Cardillo A., Scellato S., Latora V., Porta S. (2006). Structural properties of planar graph of urban street patterns. Phys. Rev. E.

[5] Newman M. (2001). Scientific collaboration networks I: Network construction and fundamental results. Phys. Rev. E.

[6] Guelzim N., Bottani S., Bourgine P. (2002). Topological and causal structure of the yeast transcriptional regulatory network. Nat. Genet..

[7] Su H., Yang C., Ferrigno G., Momi E. (2019). Improved human-robot collaborative control of redundant robot for teleoperated minimally invasive surgery. IEEE Robot. Autom. Lett..

[8] Qi W., Ovur S., Li Z., Marzullo A., Song R. (2021). Multi-sensor guided hand gestures recognition for teleoperated robot using recurrent neural network. IEEE Robot. Autom. Lett..

[9] Qi W., Su H., Aliverti A. (2020). A smartphone-based adaptive recognition and real-time monitoring system for human activities. IEEE Trans. Hum.-Mach. Syst..

[10] Ruan X., Ma L., Zhang Y., Wang Q., Gao X. (2021). Dissection of the complex transcription and metabolism regulation networks associated with maize resistance to ustilago maydis. Entropy.

[11] Strogatz S., Stewart I. (1993). Coupled oscillators and biological synchronization. Sci. Am..

[12] Gray C. (1994). Synchronous oscillations in neuronal systems: Mechanisms and functions. J. Comput. Neurosci..

[13] Wang R., Chen L. (2005). Synchronizing genetic oscillators by signaling molecules. J. Biol. Rhythm..

[14] Hu C., He H., Jiang H. (2021). Edge-based adaptive distributed method for synchronization of intermittently coupled spatiotemporal networks. IEEE Trans. Autom. Control.

[15] Liu M., Jiang H., Hu C., Yu Z., Li Z. (2020). Pinning synchronization of complex delayed dynamical networks via generalized intermittent adaptive control strategy. Int. J. Robust Nonlinear Control.

[16] Liu M., Jiang H., Hu C. (2016). Synchronization of hybrid-coupled delayed dynamical networks via aperiodically intermittent pinning control. J. Frankl. Inst..

[17] Chen C., Li L., Peng H., Yang Y. (2018). Adaptive synchronization of memristor-based BAM neural networks with mixed delays. Appl. Math. Comput..

[18] Li J., Jiang H., Hu C., Alsaedi A. (2019). Finite/fixed-time synchronization control of coupled memristive neural networks. J. Frankl. Inst..

[19] Ji G., Hu C., Yu J., Jiang H. (2018). Finite-time and fixed-time synchronization of discontinuous complex networks: A unified control framework design. J. Frankl. Inst..

[20] Liu M., Jiang H., Hu C. (2018). Aperiodically intermittent strategy for finite-time synchronization of delayed neural networks. Neurocomputing.

[21] Liu M., Jiang H., Hu C. (2017). Finite-time synchronization of delayed dynamical networks via aperiodically intermittent control. J. Frankl. Inst..

[22] Polyakov A. (2012). Nonlinear feedback design for fixed-time stabilization of linear control systems. IEEE Trans. Autom. Control.

[23] Wan P., Sun D., Zhao M. (2020). Finite-time and fixed-time anti-synchronization of Markovian neural networks with stochastic disturbances via switching control. Neural Netw..

[24] Li H., Li C., Huang T., Ouyang D. (2017). Fixed-time stability and stabilization of impulsive dynamical systems. J. Frankl. Inst..

[25] Zhang W., Li H., Li C., Li Z., Yang X. (2019). Fixed-time synchronization criteria for complex networks via quantized pinning control. ISA Trans..

[26] Feng L., Hu C., Yu J., Jiang H., Wen S. (2021). Fixed-time synchronization of coupled memristive complex-valued neural networks. Chaos Solitons Fractals.

[27] Hu C., Jiang H. (2021). Special functions-based fixed-time estimation and stabilization for dynamic systems. IEEE Trans. Syst. Man Cybern. Syst..

[28] Wang Z., Wu H. (2019). Global synchronization in fixed time for semi-Markovian switching complex dynamical networks with hybrid couplings and time-varying delays. Nonlinear Dyn..

[29] Cao J., Li R. (2017). Fixed-time synchronization of delayed memristor-based recurrent neural networks. Sci. China Inf. Sci..

[30] Chen C., Li L., Peng H., Yang Y. (2019). Fixed-time synchronization of inertial memristor-based neural networks with discrete delay. Neural Netw..

[31] Wang L., Zeng Z., Hu J., Wang X. (2017). Controller design for global fixed-time synchronization of delayed neural networks with discontinuous activations. Neural Netw..

[32] Hu C., He H., Jiang H. (2021). Fixed/preassigned-time synchronization of complex networks via improving fixed-time stability. IEEE Trans. Cybern..

[33] Hu C., Jiang H. (2015). Pinning synchronization for directed networks with node balance via adaptive intermittent control. Nonlinear Dyn..

[34] Liu M., Yu Z., Jiang H., Hu C. (2017). Synchronization of complex networks with coupled and self-feedback delays via aperiodically intermittent strategy. Asian J. Control.

[35] Gong S., Guo Z., Wen S., Huang T. (2021). Finite-time and fixed-time synchronization of coupled memristive neural networks with time delay. IEEE Trans. Cybern..

